# Reply to Lev-Ari: Operational definition of “color perception”; which task represents the concept better?

**DOI:** 10.1073/pnas.2026301118

**Published:** 2021-02-22

**Authors:** Arash Afraz, Maryam Hasantash

**Affiliations:** ^a^National Institute of Mental Health, NIH, Bethesda, MD 20892;; ^b^School of Cognitive Sciences, Institute for Research in Fundamental Sciences, 19568364 Tehran, Iran

This is the response to the Letter to the Editor by Shiri Lev-Ari (1), whom we thank for formulating a thoughtful critique of our work (2). In fact, we have been preoccupied with similar questions throughout the design stages of the study, and we are happy to explain why we chose simple color matching as our operational definition of color perception, as opposed to reaction time measures.

The ability to perceive color can be defined in different ways: How fast do we categorize color? How quickly do we notice an odd color among others? How accurately can we match colors? The term “color perception,” like any other natural concept, is loosely defined and has been related to any of these measures. The mere fact that linguistic color boundaries have been shown in some (and not all) cases to affect reaction times (3) is informative and important but does not justify using them as the basic measure of “color perception.” Obviously, we are not searching for a task that shows an effect; we are looking for a task that represents the concept best.

When asked to make speeded responses, many brain subsystems (sensory and motor cortices, basal ganglia, memory-related structures, attention-related resources, etc.) need to coordinate in real time to perform the task. Failure in any component of this coordination translates into a reaction time cost, and thus makes it a sensitive psychophysical task. However, this is not the type of sensitivity we want, as it is not specific enough to “color perception” in its most primary sense. Instead, we want to relax the fast coordination requirement for other subsystems and find a task that is bottlenecked only by the ability to discern colors no matter how long it takes. Color matching task is the basis for development of color spaces (4) and has been used to measure subtle perceptual effects (5). Therefore, we chose color matching as our operational definition of color perception, which is in fact very sensitive to potential warping of the color space. Now, is this task too easy, suffering from a ceiling effect? The size of the perceptual error is multiple (∼6) times bigger than the grain of measurement here; also, the distribution of errors stands well above zero. So, psychophysical performance is not saturated. It is notable, though, that because this part of our findings is a null result, we cannot refute the existence of a possible undetected effect of language. Nevertheless, the upper bound on the size of such a potential effect is constrained by our measurements to a very narrow band near the limits of normal human color vision. As humble as the claim may be, we thus suffice to state that variation in color vocabulary does not significantly warp the fabric of human color space; we all “perceive” color in a more-or-less similar way. Looking at the bigger picture, it is in stark contrast with the strong link between language and color memory as shown in the results.

## References

[1] S. Lev-Ari, Richer color vocabulary is correlated with color memory, but its relation to perception is unknown. Proc. Natl. Acad. Sci. U.S.A., 10.1073/pnas.2024682118 (2021). PMC7958243 33619179

[2] M. Hasantash, A. Afraz, Richer color vocabulary is associated with better color memory but not color perception. Proc. Natl. Acad. Sci. U.S.A. 117, 31046–31052 (2020). 33229567 10.1073/pnas.2001946117 PMC7733847

[3] J. A. Winawer, N. Witthoft, L. Wu, L. Boroditsky, Effects of language on color discriminability. J. Vis. 3, 711 (2003).

[4] A. D. Broadbent, A critical review of the development of the CIE1931 RGB color-matching functions. Color Res. Appl. 29, 267−272 (2004).

[5] E. H. Land, The retinex theory of color vision. Sci. Am. 237, 108–128 (1977). 929159 10.1038/scientificamerican1277-108

